# A Metabolomics Approach to Sulforaphane Efficacy in Secondhand Smoking-Induced Pulmonary Damage in Mice

**DOI:** 10.3390/metabo12060518

**Published:** 2022-06-03

**Authors:** Hongyoon Kim, Sunju Yoo, Jung-Dae Lee, Hyang-Yeon Kim, Suhkmann Kim, Kyu-Bong Kim

**Affiliations:** 1 College of Pharmacy, Dankook University, 119 Dandae-ro, Cheonan 31116, Korea; hongyoon1@naver.com (H.K.); ljd0734@nate.com (J.-D.L.); festivalkim@naver.com (H.-Y.K.); 2 Center for Human Risk Assessment, Dankook University, 119 Dandae-ro, Cheonan 31116, Korea; 3 College of Natural Sciences, Dankook University, 119 Dandae-ro, Cheonan 31116, Korea; sunju408@naver.com; 4 Department of Chemistry and Chemistry Institute for Functional Materials, Pusan National University, Busan Daehak-ro 63beon-gil 2, Busan 46241, Korea; suhkmann@pusan.ac.kr

**Keywords:** NMR, metabolite, pulmonary disease, sulforaphane, second-hand smoke

## Abstract

Sulforaphane is an isocyanate abundantly present in cruciferous vegetables. In the present study, we aimed to investigate the effects of sulforaphane on secondhand smoking (SHS)-induced pulmonary damage in mice. Additionally, a metabolomic study was performed to identify biomarkers associated with pulmonary disease using proton nuclear magnetic resonance (^1^H-NMR) analysis. Male C57BL6J mice were divided into a control group, an SHS exposure group (positive control group, PC), and a sulforaphane treatment group exposed to secondhand smoke (SS) (*n* = 5 per group). The PC and SS groups were exposed to secondhand smoke in a chamber twice daily for four weeks. Mice in the SS group were orally administered sulforaphane (50 mg/kg) for four weeks during secondhand smoke exposure. Histopathological examination of the lungs revealed pulmonary damage in PC mice, including loss of bronchial epithelial cells, bronchial wall thickening, and infiltration of macrophages. In contrast, mice in the SS group showed little or no epithelial thickening, thereby exhibiting reduced lung damage. Mouse serum and lung tissues were collected and analyzed to determine changes in endogenous metabolites using ^1^H-NMR. After target profiling, we identified metabolites showing the same tendency in the serum and lung as biomarkers for SHS-induced pulmonary damage, including taurine, glycerol, creatine, arginine, and leucine. As a result of histopathological examination, sulforaphane might inhibit SHS-induced lung damage, and metabolite analysis results suggest potential biomarkers for SHS-induced pulmonary damage in mice.

## 1. Introduction

Sulforaphane is an isocyanate compound abundantly present as its precursor, glucoraphanin, in cruciferous vegetables, such as cabbage, broccoli, and kale [1,2]. Raw broccoli reportedly contains 0.005–1.13 μmol/g glucoraphanin, which is converted into sulforaphane by myrosinase. Myrosinase is not produced in mammals; therefore, microorganisms in the gastrointestinal tract metabolize glucoraphanin to sulforaphane. Sulforaphane acts as a phase II enzyme inducer in the body to induce the activities of NAD(P)H: quinone reductase (NQO1) and glutathione-S-transferases. In addition, sulforaphane can activate nuclear factor-erythroid factor 2-related factor 2 (Nrf2), enhancing its biological functions, such as anti-inflammatory and antioxidant activities [3,4]. Given that glucoraphanin conversion markedly varies (metabolite excretion was 1%–45% of intake) [1], sulforaphane was directly used in the present study. Sulforaphane increases the expression of the p53 gene, which reportedly induces apoptosis in cancer cells, such as liver and cervical cancer cells, maintaining cells in the sub-G1 phase [5]. In autoimmune encephalomyelitis or pulmonary adenoma-induced mice, sulforaphane treatment was shown to exhibit antioxidant effects, decrease the inflammatory response, and suppress the malignant progression of pulmonary adenoma [6,7].

The lungs are essential organs, as well as important sites of various diseases, including idiopathic pulmonary fibrosis (IPF) and chronic obstructive pulmonary disease (COPD). In particular, IPF and COPD are growing global health challenges, primarily attributed to smoking habits. Currently, no available therapy can reduce the inevitable progression of these diseases [2,8,9,10,11]. Notably, only one in four patients with COPD in the United States was found to be a non-smoker, and eight out of ten COPD-related deaths occurred among smokers (US-DHHS, 2014). In addition, non-smokers with COPD were more likely to have experienced long-term exposure to lung irritants, such as secondhand smoke. Therefore, cigarettes have been employed in models of pulmonary injury. Moreover, the intensity of lung damage induced by indirect smoking has been previously examined [12,13].

Metabolomics has been used for elucidating biomarkers, mechanisms of diseases or biological responses caused by various factors through analysis of endogenous metabolites of low molecular weight (100–1000 Da) [14,15]. Metabolomics has been used to study in vivo metabolic reactions to external stressors and develop diagnostic and prognostic biomarkers of human diseases or chemical-induced toxicities [16,17,18]. It primarily uses nuclear magnetic resonance (NMR) spectroscopy and mass spectrometry. In the present study, samples were analyzed using proton NMR (^1^H-NMR), which can simultaneously measure several metabolites, and samples can be recovered and reused in other analytical instruments. Therefore, ^1^H-NMR has been utilized in several research fields to identify biomarkers [19,20,21,22].

Accordingly, in the current study, we aimed to demonstrate the effects of sulforaphane on secondhand smoking (SHS)-induced pulmonary damage in mice. In addition, a metabolomic study was performed to identify mechanisms or biomarkers related to pulmonary disease using ^1^H-NMR analysis.

## 2. Results

### 2.1. Changes in Body Weights

**Figure 1:**
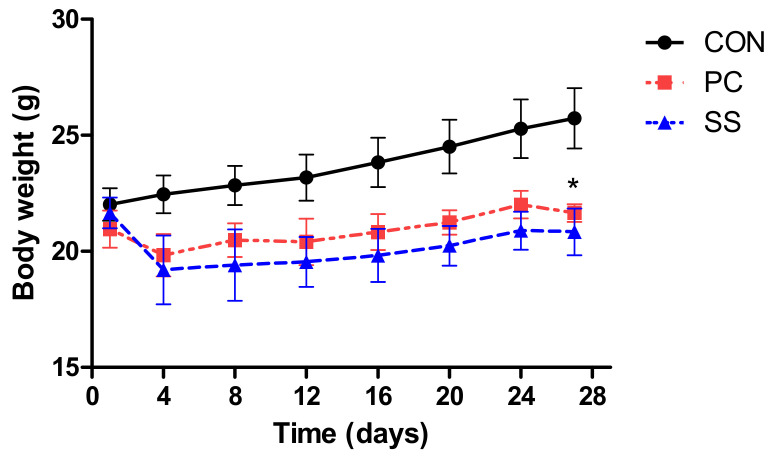
The body weight curves of each group. The body weight curves of positive control (PC) and test (SS) were likely to be decreased compared to control group (CON). (*n* = 5, *, *p* < 0.05).

Compared with the CON group, mice in the PC and SS groups experienced initial body weight loss following SHS exposure. Weight loss in both PC and SS was observed after four days of exposure, followed by a gradual increase in body weight in both groups (Figure 1).

### 2.2. Histopathologic Examination

**Figure 2:**
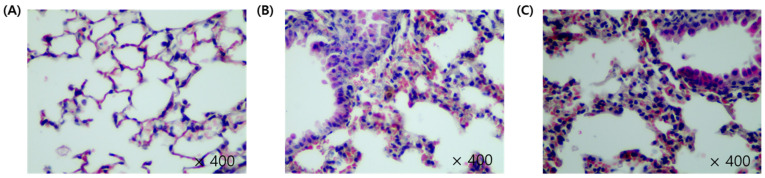
Histopathological analysis of the lungs in C57BL/6J mice. The lung sections were stained with haemotoxylin and eoxin(H&E), ×400. (**A**) The lung image of control (CON), (**B**) Positive control (PC), (**C**) and test (SS) group.

Type 1 and type 2 pulmonary epithelial cells were identified in the CON group, along with normal alveolar macrophages (Figure 2). The PC group exhibited loss of bronchial epithelial cells, accompanied by lesions with thickened cell walls. In addition, bronchioles showed the presence of cell debris, while macrophages displayed yellow and black tar particles. Tar particles were also detected in SS mice; however, only a small number of macrophages contained tar. Mice in the SS group showed minimal or no epithelial thickening. Some mice demonstrated proliferation of type 2 pulmonary epithelial cells; however, the damage was not significant when compared with the PC group (Figure 2).

### 2.3. SHS Regulated Metabolites in Serum and Lungs

**Figure 3:**
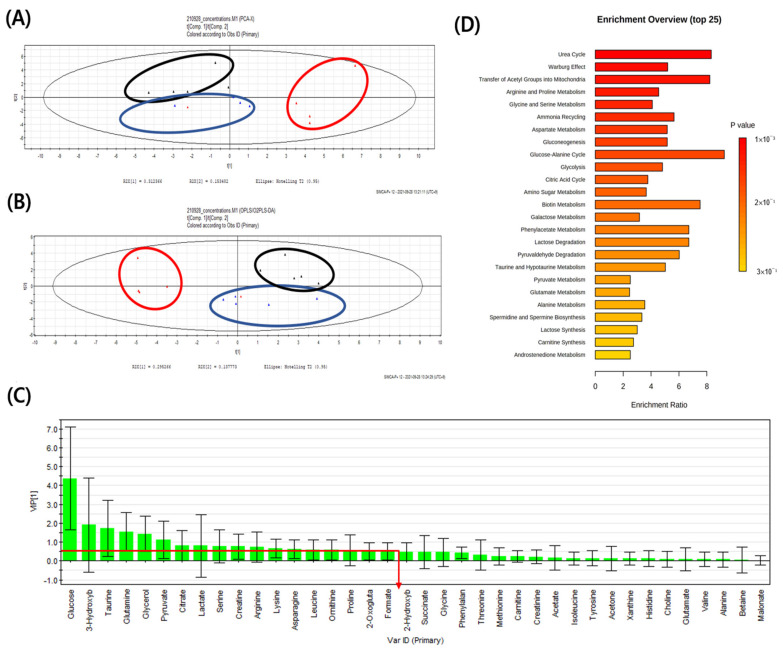
A comparison of metabolite patterns of serum using ^1^H-NMR spectroscopy. (**A**) Principal component analysis (PCA) (R^2^X = 1, Q^2^ = 0.999) and (**B**) orthogonal projections to latent structures-discriminant analysis (OPLS-DA) (R^2^X = 0.707, Q^2^ = 0.478) models results after NMR analysis of control, positive control, and test group in serum samples. (**C**) Variable importance plot (VIP) shows the major serum metabolites that contributed to separate the clusters (VIP ≥ 0.5) and (**D**) Metabolites set enrichment overview (*n* = 5); ▲, control (CON); 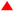, positive control (PC); 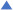, test (SS).

**Figure 4:**
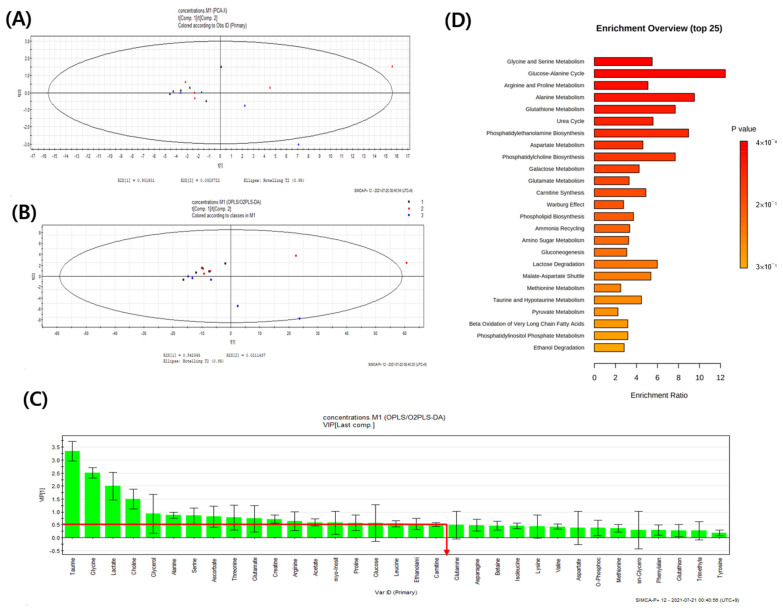
Pattern recognition of lung metabolomics profiles using ^1^H-NMR spectroscopy. (**A**) Principal component analysis (PCA) (R^2^X = 0.997, Q^2^ = 0.903) and (**B**) orthogonal projections to latent structures-discriminant analysis (OPLS-DA) (R^2^X = 0.963, Q^2^ = 0.201) models results after NMR analysis of control, positive control and test group in serum samples. (**C**) Variable importance plot (VIP) shows the major serum metabolites that contributed to separate the clusters (VIP ≥0.5) and (**D**) Metabolites set enrichment overview (*n* = 5); ▲, control (CON); 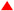, positive control (PC); 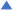, test (SS).

Serum and lung sample ^1^H-NMR spectra of CON, PC, and SS groups were obtained. The spectral region of δ 0.0–10.0 was segmented into regions of 0.04 ppm width, providing 250 integrated regions in each ^1^H-NMR spectrum for serum and lung samples. Visual examination of ^1^H-NMR spectra revealed the distinct intensities of some metabolites between groups. Spectral binning data were obtained through ^1^H-NMR analysis of the mouse serum and lung samples. Global profiling of serum did not show separated clustering in PCA using SIMCA-P multivariate analysis, but these profiles were notably separated in OPLS-DA (Figure S2). Global profiling of the lung tissue did not show separated clustering in the PCA and OPLS-DA analyses (Figure S3). In PCA, target profiling failed to reveal clear clustering of both serum and lung tissues; however, a clear clustering was observed in serum OPLS-DA. No clear clustering was observed in OPLS-DA of lung tissue (Figure 3A,B and Figure 4A,B).

**Figure 5:**
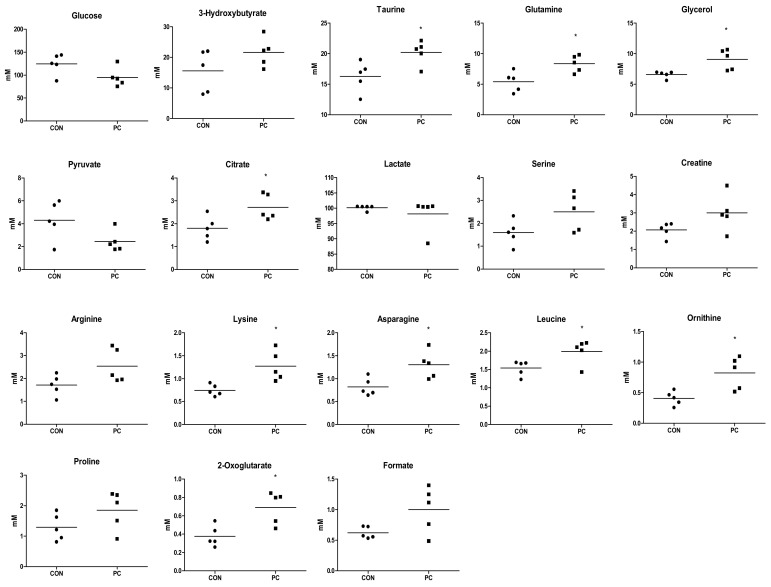
Concentrations of endogenous metabolites in serum samples of mice. Major serum metabolites (variable importance plot (VIP) ≥0.5), *t*-test and Unpaired *t*-test were performed to assess statistical significance compared with control (CON) and positive control (PC); ●, CON; ■, PC; *, *p* < 0.05.

In total, 38 endogenous metabolites were identified in serum samples using the Chenomx NMR Suite program. PCA and OPLS-DA showed clustering separated by metabolic patterns in CON, PC, and SS groups on metabolite analysis of serum samples. (Figure 3A,B). Metabolites with a VIP value of ≥0.5 were selected as meaningful metabolites for CON and PC classification (Figure 3C). Overall, 18 serum metabolites were selected, including glucose, 3-hydroxybutyrate, taurine, glutamine, glycerol, pyruvate, citrate, lactate, serine, creatine, arginine, lysine, asparagine, leucine, ornithine, proline, 2-oxoglutarate, and formate. Except for glucose, pyruvate and lactate, levels of metabolites were higher in the PC group than in the CON group (Figure 5). Statistical significance (*p* < 0.05) was confirmed for taurine, glutamine, glycerol, citrate, lysine, asparagine, leucine, ornithine, and 2-oxoglutarate.

**Figure 6:**
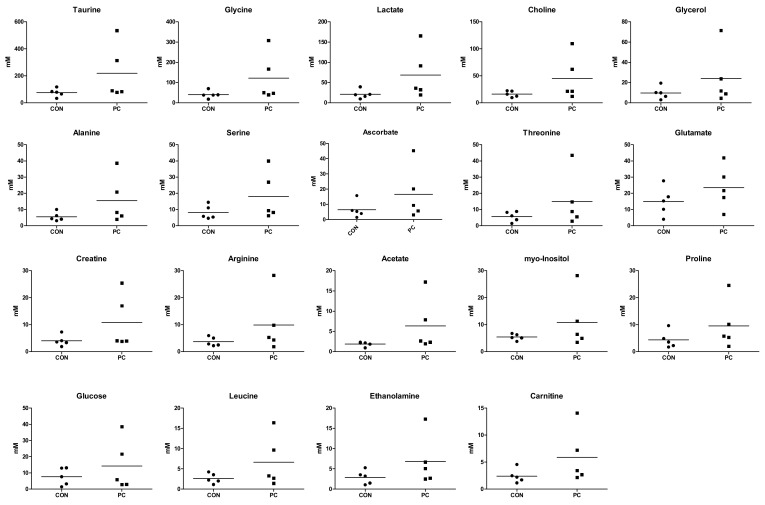
Concentrations of endogenous metabolites in lung samples of mice. Major lung metabolites (variable importance plot (VIP) ≥0.5), *t*-test and Unpaired *t*-test were performed to assess statistical significance compared with control (CON) and positive control (PC); ●, CON; ■, PC.

In total, 33 endogenous metabolites were identified in the lungs. Score plots of PCA and OPLS-DA did not show clear clustering of metabolic patterns among the three groups (Figure 4A,B). VIP sorted endogenous metabolites based on their contribution to clustering separation (Figure 4C). Metabolites with a VIP value of ≥0.5 were selected as meaningful metabolites for classifying each group (Figure 4C). Overall, 19 lung metabolites were selected, including taurine, glycine, lactate, choline, glycerol, alanine, serine, ascorbate, threonine, glutamate, creatine, arginine, acetate, myo-inositol, proline, glucose, leucine, ethanolamine, and carnitine. Compared with the CON group, all metabolites exhibited the tendency of increasing levels in the PC group although the differences between CON and PC are not statistically significant (Figure 6).

## 3. Discussion

SHS can be divided into two main categories: direct inhalation of the smoker’s exhalation and sidestream smoke that spreads through the air with cigarette burning [23]. Herein, the effects of sulforaphane were examined on SHS-induced pulmonary injury.

Nicotine acutely increases energy expenditure and can induce acute anorexic effects [24]. Accordingly, a reduction in body weight was observed within four days of exposure in the PC and SS groups exposed to cigarette smoke. Moreover, weight loss attributed to SHS is likely not recovered by sulforaphane treatment, consistent with previous studies [25].

In the present study, the PC group displayed type 2 cell proliferation and thickened cell walls when compared with the CON group (Figure 2). Type 1 pulmonary epithelial cells cover >95% of the alveolar surface and play a role in gas exchange between the alveoli and blood. Type 2 cells secrete pulmonary surfactants to reduce the surface tension and facilitate gas exchange. In lungs exposed to toxic substances, type 1 cells fail to undergo replication and are susceptible to toxicity, whereas type 2 cells are capable of proliferation. Therefore, type 2 cell proliferation in the PC group indicates the occurrence of lung damage [26]. Although these symptoms were observed in the SS group, they were not significant compared with the CON group. Macrophages and tar were detected in the lungs of SS mice, but the damage was minimal when compared with PC mice. In addition, little or no epithelial thickening was observed in the SS group. SHS-induced pulmonary damage was observed in PC mice, and preventive effects of sulforaphane on pulmonary lung damage were observed in the SS group compared with the PC group. However, given that sulforaphane was administered orally, short-term administration failed to exhibit significant efficacy.

**Figure 7:**
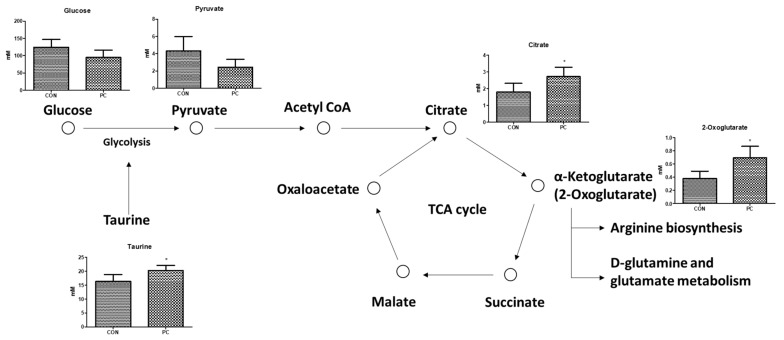
Expected serum metabolic pathway by second-hand smoke exposure. Error bars are expressed as S.D. * *p* < 0.05.

**Figure 8:**
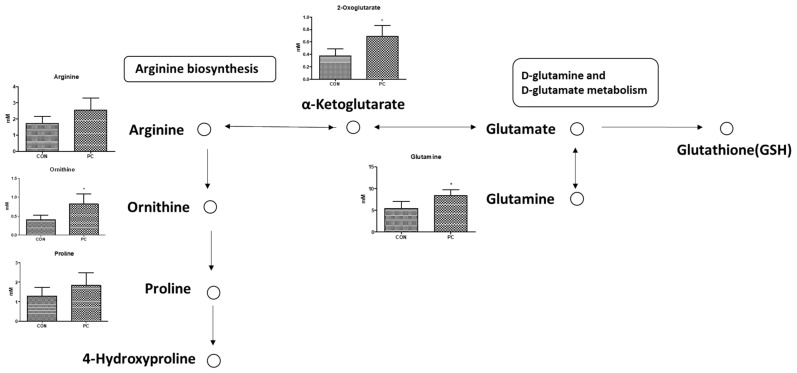
Expected serum metabolic pathway by second-hand smoke exposure. Error bars are expressed as S.D. *, *p* < 0.05.

The present metabolomics study was compared with metabolites determined in previous studies to identify biomarkers for the pulmonary injury model (Figure 7 and Figure 8). Compared with CON mice, PC mice exhibited increased levels of glycerol and taurine in the serum and lungs (Figure 5 and Figure 6). During inflammatory diseases, enhanced metabolic consumption of lipids leads to increased ketone bodies and glycerol levels [27]. Increased glycerol levels in the PC group could be attributed to inflammation-induced fat metabolism. Taurine, either dietary or injected, reportedly exerts anti-inflammatory effects [28,29,30,31]. In addition, taurine is abundantly present in the cytoplasm of neutrophils and can protect tissues at the inflammatory site [32,33]. Given these properties, elevated taurine levels in the serum and lungs of PC mice were potentially due to SHS-induced inflammation. Serum glucose levels tend to decrease during lung diseases such as cystic fibrosis [34]. Therefore, reduced serum glucose levels in the PC group appear to be related to pulmonary injury. Reduced serum glucose levels can result in decreased pyruvate levels. However, given elevated acetyl CoA levels following enhanced lipid metabolism, citrate, a product of the tricarboxylic acid cycle, was reportedly increased. α-Ketoglutarate, produced by the breakdown of citrate in the serum, can generate glutamine or arginine. It has been reported that patients with acute COPD exhibit elevated serum levels of arginine, and pulmonary damage caused by inhalation or oxidative stress increases arginase activity, elevating hydroxyproline levels and inducing collagen synthesis [35,36]. Herein, arginine, ornithine, and proline levels were increased in PC mice. In addition, elevated glutamine levels were observed in PC serum, inducing the synthesis of glutathione (GSH), an antioxidant [37]. Our findings confirmed that pulmonary damage was increased in the PC group when compared with the CON group (Figure 7 and Figure 8). Accordingly, an SHS-induced lung injury model can be established by confirming changes in serum metabolites as described above.

Subsequently, we compared lung and serum metabolites and noted that glucose, taurine, glycerol, lactate, serine, creatine, arginine, leucine, and proline were metabolites with VIP ≥ 0.5 in both serum and lung samples (Figure 5 and Figure 6). After four weeks of exposure to SHS, metabolite changes in the lungs were similar, except for glucose and lactate levels. Although serum glucose was decreased, lung glucose levels were increased, owing to enhanced glycolysis during inflammation [37,38]. Serum lactate levels did not show significant differences between groups, and lung serine and proline levels did not differ between PC and SS. Given these metabolite differences between serum and lung samples in the present study, taurine, glycerol, creatine, arginine, and leucine, all metabolites with VIP ≥ 0.5 in both serum and lung, were suggested as biomarkers exhibiting the same tendency.

**Figure 9:**
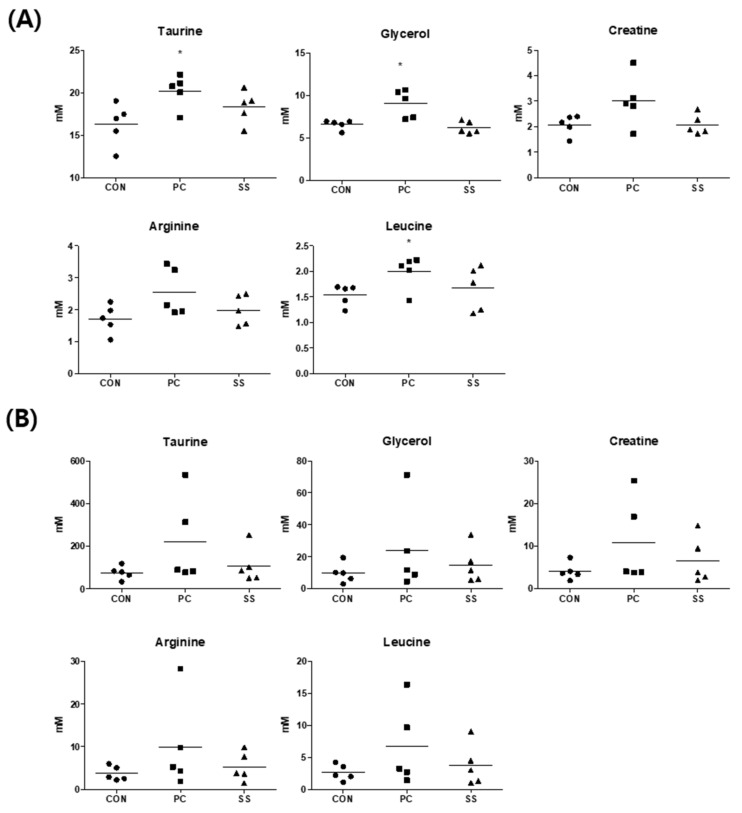
Concentrations of endogenous metabolites of mice. (**A**) The metabolite change of serum samples and (**B**) lung samples. One-way ANOVA test and Tukey’s test were performed to assess statistical significance compared with control (CON), positive control (PC) and test (SS); ●, CON; ■, PC; ▲, SS; Error bars are expressed as S.D. *, *p* < 0.05.

Histopathological examination confirmed that oral sulforaphane (50 mg/kg) exerted preventive effects in an SHS-induced mouse model. In addition, the use of biomarkers in the mouse model confirmed the changes in SS metabolites. All biomarkers in the SS group, showed a tendency for higher levels than those in CON mice and lower levels than those in PC mice (Figure 9). In the SS group, taurine, glycerol, and arginine, all indicators of inflammation, were higher than in the CON group and lower than those in the PC group. Statistical significance was not confirmed in all metabolites of lung samples and creatine and arginine in serum samples, but the same trend was confirmed in both samples and this was suggested as a biomarker. Based on our findings, sulforaphane has an inhibitory effect on pulmonary injury, and SHS induces notable biomarkers.

## 4. Methods

### 4.1. Chemicals

Sulforaphane was purchased from LKT Laboratories, Inc. (St. Paul, MN, USA) and 3-(trimethylsilyl) propionic-2,2,3,3-d4 acid sodium salt (TSP-d_4_) were obtained from Sigma-Aldrich (St. Louis, MO, USA).

### 4.2. Animals and Treatment

This study was approved by the Institutional Animal Care and Use Committee of Dankook University (approval number, 20-027). C57BL/6J mice (6-weeks-old) were purchased from Samtako Bio Korea (Osan, Gyeonggido, Korea). The animals underwent acclimatization for one week under standard conditions, with a 12-h light/dark cycle at 22 °C and 55 ± 5% relative humidity. Food (standard diet, Samtako Bio, Osan, Korea) and tap water were provided ad libitum. Experimental animals were divided into a control group (CON), an SHS exposure group (positive control, PC), and a sulforaphane treatment group exposed to SHS (test, SS), and each group consisted of five animals. The body weight of the mice was measured daily at the same time for four weeks.

PC and SS groups were simultaneously exposed to cigarette smoke (six cigarettes; THIS ORIGINTM, KT&G Inc., Daejeon, Korea) twice daily and six times/week for four weeks using a custom-designed acrylic chamber (39.5 × 37.5 × 42 cm, Figure S1). Each exposure lasted for 75 min.

Following exposure to cigarette smoke, animals in the SS group were orally administered 50 mg/kg of sulforaphane dissolved in 0.9% sodium chloride solution six times/week for four weeks. At the end of the animal study, mice were euthanized with CO_2_, and blood was collected from the abdominal aorta. After incubation for 1 h at room temperature, the serum was separated by centrifugation at 13,000× *g* for 15 min. After blood collection, the lungs were excised and divided into two parts: one part was fixed in 10% formalin for histopathological examination; the other was immediately frozen in liquid nitrogen for metabolomics. The lungs were not perfused. Serum and lung samples were stored at −80 °C before ^1^H-NMR analysis.

### 4.3. Histopathologic Examination

After tying one side of the bronchus and cutting half of the lung to prevent formalin entry, 10% (*v*/*v*) neutral-buffered formalin was injected through the airway for tissue fixation. After 24 h, the tissue was dehydrated, embedded in paraffin, and cut into 4 μm sections. The sections were then stained with hematoxylin and eosin and examined under a light microscope (Eclipse Ci series, Nikon, Tokyo, Japan).

### 4.4. Metabolomics

#### 4.4.1. H-NMR Spectroscopic Analysis

Both lung and serum samples were analyzed using ^1^H-NMR spectroscopy. Sample types were different, therefore, we optimized pulse parameters before NMR measurements for each lung and serum sample. Samples were thawed prior to analysis and stored at 4 °C. All ^1^H-NMR spectra were acquired using a 600 MHz high-resolution magic angle spinning (HR-MAS) NMR spectrometer, equipped with a 4-mm gHX NanoProbe (Agilent Technologies, Santa Clara, CA, USA). The spinning rate was set at 2050 Hz, and the Carr–Purcell–Meiboom–Gill (CPMG) pulse sequence was applied for macromolecule and water signal suppression. Water resonance was suppressed by offset frequency of 4.8 ppm with 79 Hz presaturation power. The measurements were performed at 298 K. Lung samples (20 mg) were weighed and placed in ^1^H-NMR nanotubes (Agilent Sample Tube, 4 mm). A total of 20 μL phosphate buffer (pH 7.4) in deuterated water (D_2_O) containing 2 mM 3-trimethylsilyl-2,2,3,3-tetradeuteropropionic acid-d4 (TSP-d_4_, Sigma-Aldrich, St. Louis, MO, USA) was added to the nanotubes. TSP-d_4_ was used as an internal chemical shift standard. For the lung tissue analysis, acquisition time was set at 3 s, 9.06 μs 90-degree pulse (pw), 3 s relaxation delay, 500 μs spin-echo delay with 150 duty cycles; the total acquisition time was 13 min 20 s.

For serum sample analysis, 36 μL of serum was mixed with 4 μL of phosphate-buffered saline in D_2_O containing 20 mM TSP-d_4_ and placed in a ^1^H-NMR nanotube (Agilent Sample Tube, 4 mm). Serum ^1^H-NMR spectra were measured using a 3 s acquisition time, 8.85 μs pw, 1.5 s relaxation delay, 450 μs spin-echo delay with 166 duty cycle; the total acquisition time was 11 min 12 s. In total, 128 scans were acquired for each sample at a spectral width of 24,038.5 Hz.

^1^H-NMR spectra were processed using the Chenomx NMR Suite program (ver. 8.3, Chenomx Inc., Edmonton, Alberta, Canada). The δ 0.0–10.0 spectral region was segmented into regions of 0.04 ppm width, providing 250 integrated regions in each ^1^H-NMR spectrum. This binning process endowed each segment with an integral value, providing an intensity distribution of the entire spectrum with 250 variables prior to the pattern recognition analysis. The spectrum region of water (δ 4.5–5.0) was removed from the analysis to prevent variations in water suppression efficiency. Spectra were identified and quantified using the Chenomx NMR Suite Professional software package, ver. 8.3 (Chenomx, Inc.). The information of peaks used for identification of endogenous metabolites is listed in Table S1. TSP-d_4_ (2 mM for lung and 2 mM for serum) was used as standard to measure relative metabolite concentrations and normalize samples. Before applying targeted profiling to CPMG spectra in Chenomx, we adjusted the apparent linewidth of the chemical shape indicator (CSI) following Chenomx tutorial user guide using glucose and TSP-d_4_ peaks.

#### 4.4.2. Multivariate and Statistical Analysis

Data were converted from Microsoft Excel (*.xls) to the NMR Suite Professional software format. One-dimensional NMR spectral data were imported into SIMCA-P (version 12.0, Umetrics Inc., Kinnelon, NJ, USA) to examine intrinsic variations in the dataset for multivariate statistical analysis. Pareto was used to scale the data before principal component analysis (PCA) and orthogonal projections to latent structure discriminant analysis (OPLS-DA). Variable importance plots (VIP) were also used to select putative biomarkers for SHS-induced pulmonary damage.

Serum and lung metabolite concentrations were statistically analyzed using ANOVA and Tukey’s test (GraphPad Prism 5, San Diego, CA, USA). Statistical significance was set at *p* < 0.05.

## 5. Conclusions

The current results demonstrated that exposure to SHS for four weeks could induce minor pulmonary injury. Oral administration of sulforaphane could reduce SHS-induced lung tissue damage and decrease epithelial cell dropout in bronchioles. We noted that five metabolites in the PC serum and lungs showed similar tendencies. Accordingly, taurine, glycerol, arginine, and proline, exhibiting the same changes in serum and lung tissue metabolites, were identified as biomarkers for SHS-induced lung injury. In addition, the use of plasma biomarkers can reduce animal sacrifice via the noninvasive identification of animal models.

## Data Availability

The data presented in this study are available on request from the corresponding author. Those data are belong to the institute. The data will be provided only after permission from the institute of Dankook University.

## Supplementary Materials

The followings are available online at https://www.mdpi.com/article/10.3390/metabo12060518/s1, Figure S1: Custom-designed acrylic chamber (39.5 × 37.5 × 42 cm), Figure S2: A comparison of serum binning patterns using ^1^H-NMR spectroscopy, Figure S3: A comparison of lung binning patterns using ^1^H-NMR spectroscopy, Table S1: The information of peaks used for identification of metabolites in serum (S) and lung (L).

## References

[1] Yagishita Y., Fahey J.W., Dinkova-Kostova A., Kensler T.W. (2019). Broccoli or Sulforaphane: Is It the Source or Dose That Matters?. Molecules.

[2] Zhao Y.D., Yin L., Archer S., Lu C., Zhao G., Yao Y., Wu L., Hsin M., Waddell T.K., Keshavjee S. (2017). Metabolic heterogeneity of idiopathic pulmonary fibrosis: A metabolomic study. BMJ Open Respir. Res..

[3] Houghton C.A., Fassett R.G., Coombes J.S. (2016). Sulforaphane and Other Nutrigenomic Nrf2 Activators: Can the Clinician’s Expectation Be Matched by the Reality?. Oxidative Med. Cell. Longev..

[4] Nguyen T., Huang H.C., Pickett C.B. (2000). Transcriptional regulation of the antioxidant response element: Activation by Nrf2 and repression by MafK. J. Biol. Chem..

[5] Park S.Y., Bae S.-J., Choi Y.H. (2005). Anti-proliferative Effects of the Isothiocyanate Sulforaphane on the Growth of Human Cervical Carcinoma HeLa Cells. J. Life Sci..

[6] Conaway C.C., Wang C.-X., Pittman B., Yang Y.-M., Schwartz J.E., Tian D., McIntee E.J., Hecht S.S., Chung F.-L. (2005). Phenethyl Isothiocyanate and Sulforaphane and their *N*-Acetylcysteine Conjugates Inhibit Malignant Progression of Lung Adenomas Induced by Tobacco Carcinogens in A/J Mice. Cancer Res..

[7] Yoo I.H., Kim M.J., Kim J., Sung J.J., Park S.T., Ahn S.W. (2019). The Anti-Inflammatory Effect of Sulforaphane in Mice with Experimental Autoimmune Encephalomyelitis. J. Korean Med. Sci..

[8] Barnes P.J., Shapiro S.D., Pauwels R.A. (2003). Chronic obstructive pulmonary disease: Molecular and cellular mechanisms. Eur. Respir. J..

[9] Harting J.R., Gleason A., Romberger D.J., Von Essen S.G., Qiu F., Alexis N., Poole J.A. (2012). Chronic Obstructive Pulmonary Disease Patients Have Greater Systemic Responsiveness to Ex Vivo Stimulation with Swine Dust Extract and its Components Versus Healthy Volunteers. J. Toxicol. Environ. Health Part A.

[10] Kang Y.P., Lee S.B., Lee J.-M., Kim H.M., Hong J.Y., Lee W.J., Choi C.W., Shin H.K., Kim D.-J., Koh E.S. (2016). Metabolic Profiling Regarding Pathogenesis of Idiopathic Pulmonary Fibrosis. J. Proteome Res..

[11] Kim K.-B., Um S.Y., Chung M.W., Jung S.C., Oh J.S., Kim S.H., Na H.S., Lee B.M., Choi K.H. (2010). Toxicometabolomics approach to urinary biomarkers for mercuric chloride (HgCl_2_)-induced nephrotoxicity using proton nuclear magnetic resonance (1H NMR) in rats. Toxicol. Appl. Pharmacol..

[12] Beckett E.L., Stevens R.L., Jarnicki A.G., Kim R.Y., Hanish I., Hansbro N.G., Deane A., Keely S., Horvat J.C., Yang M. (2013). A new short-term mouse model of chronic obstructive pulmonary disease identifies a role for mast cell tryptase in pathogenesis. J. Allergy Clin. Immunol..

[13] Hwang J., Chung S., Sundar I.K., Yao H., Arunachalam G., McBurney M.W., Rahman I. (2010). Cigarette smoke-induced autophagy is regulated by SIRT1–PARP-1-dependent mechanism: Implication in pathogenesis of COPD. Arch. Biochem. Biophys..

[14] Kim K., Lee B.M. (2009). Metabolomics, a new promising technology for toxicological research. Toxicol. Res..

[15] Roberts M.J., Schirra H., Lavin M.F., Gardiner R.A. (2014). NMR-based metabolomics: Gobal analysis of metabolites to address problems in prostate cancer. Cervical, Breast and Prostate Cancer.

[16] Ho J., Sharma A., Mandal R., Wishart D.S., Wiebe C., Storsley L., Karpinski M., Gibson I.W., Nickerson P.W., Rush D.N. (2016). Detecting renal allograft inflammation using quantitative urine metabolomics and CXCL10. Transplant. Direct.

[17] Kim S.N., Lee J., Yang H., Cho J., Kwon S., Kim Y., Her J., Cho K., Song C., Lee K. (2010). Dose-response effects of bleomycin on inflammation and pulmonary fibrosis in mice. Toxicol. Res..

[18] Ryu S.H., Lee J.D., Kim J.W., Kim S., Kim S., Kim K. (2019). ^1^H NMR toxicometabolomics following cisplatin-induced nephrotoxicity in male rats. J. Toxicol. Sci..

[19] Kim M.K., Kim K.-B., Kim H.S., Lee B.-M. (2019). Alternative skin sensitization prediction and risk assessment using proinflammatory biomarkers, interleukin-1 beta (IL-1β) and inducible nitric oxide synthase (iNOS). J. Toxicol. Environ. Health Part A.

[20] Kim H.Y., Lee Y., Kim S.J., Lee J.D., Kim S., Ko M.J., Kim J., Shin C.Y., Kim K. (2022). Metabolomics profiling of valproic acid-induced symptoms resembling autism spectrum disorders using 1H NMR spectral analysis in rat model. J. Toxicol. Environ. Health Part A.

[21] Lee J.D., Kim H.Y., Park J.J., Oh S.B., Goo H., Cho K.J., Kim S., Kim K. (2021). Metabolomics approach to biomarkers of dry eye disease using ^1^H-NMR in rats. J. Toxicol. Environ. Health Part A.

[22] Won A.J., Kim S., Kim Y.G., Kim K., Choi W.S., Kacew S., Kim K.S., Jung J.H., Lee B.M., Kim S. (2016). Discovery of urinary metabolomic biomarkers for early detection of acute kidney injury. Mol. BioSyst..

[23] Schick S., Glantz S. (2005). Philip Morris toxicological experiments with fresh sidestream smoke: More toxic than mainstream smoke. Tob. Control.

[24] Chiolero A., Faeh D., Paccaud F., Cornuz J. (2008). Consequences of smoking for body weight, body fat distribution, and insulin resistance. Am. J. Clin. Nutr..

[25] Hong H.W. (2011). The Effects of Kamgiltang on Passive Smoking in Rats. Ph.D. Thesis.

[26] Kanj R.S., Kang J.L., Castranova V. (2005). Measurement of the release of inflammatory mediators from rat alveolar macrophages and alveolar type II cells following lipopolysaccharide or silica exposure: A comparative study. J. Toxicol. Environ. Health Part A.

[27] Fitzpatrick M., Young S.P. (2013). Metabolomics—A novel window into inflammatory disease. Swiss Med. Wkly..

[28] Carneiro E.M., Latorraca M.Q., Araujo E.P., Beltrá M., Oliveras-López M.-J., Navarro M., Berna G., Bedoya F., Velloso L.A., Soria B. (2009). Taurine supplementation modulates glucose homeostasis and islet function. J. Nutr. Biochem..

[29] De la Puerta C., Arrieta F.J., Balsa J.A., Botella-Carretero J.I., Zamarrón I., Vázquez C. (2010). Taurine and glucose metabolism: A review. Nutr. Hosp..

[30] Lampson W.G., Kramer J.H., Schaffer S.W. (1983). Potentiation of the actions of insulin by taurine. Can. J. Physiol. Pharmacol..

[31] Qaradakhi T., Gadanec L.K., McSweeney K.R., Abraham J.R., Apostolopoulos V., Zulli A. (2020). The Anti-Inflammatory Effect of Taurine on Cardiovascular Disease. Nutrients.

[32] Marcinkiewicz J., Grabowska A., Bereta J., Bryniarski K., Nowak B. (1998). Taurine chloramine down-regulates the generation of murine neutrophil inflammatory mediators. Immunopharmacology.

[33] Marquez L.A., Dunford H.B. (1994). Chlorination of taurine by myeloperoxidase. Kinetic evidence for an enzyme-bound intermediate. J. Biol. Chem..

[34] Jung J., Kim S.-H., Lee H.-S., Choi G.S., Jung Y.-S., Ryu D.H., Park H.-S., Hwang G.-S. (2013). Serum metabolomics reveals pathways and biomarkers associated with asthma pathogenesis. Clin. Exp. Allergy.

[35] Sousse L.E., Yamamoto Y., Enkhbaatar P., Rehberg S.W., Wells S.M., Leonard S., Traber M.G., Yu Y.M., Cox R.A., Hawkins H.K. (2011). Acute lung injury-induced collagen deposition is associated with elevated asymmetric dimethylarginine and arginase activity. Shock.

[36] Ruzsics I., Nagy L., Keki S., Sarosi V., Illes B., Illes Z., Horvath I., Bogar L., Molnar T. (2015). L-Arginine Pathway in COPD Patients with Acute Exacerbation: A New Potential Biomarker. COPD J. Chronic Obstr. Pulm. Dis..

[37] Sappington D.R., Siegel E.R., Hiatt G., Desai A., Penney R.B., Jamshidi-Parsian A., Griffin R.J., Boysen G. (2016). Glutamine drives glutathione synthesis and contributes to radiation sensitivity of A549 and H460 lung cancer cell lines. Biochim. Biophys. Acta Gen. Subj..

[38] Corrales-Medina V.F., deKemp R.A., Chirinos J.A., Zeng W., Wang J., Waterer G., Beanlands R.S.B., Dwivedi G. (2021). Persistent Lung Inflammation After Clinical Resolution of Community-Acquired Pneumonia as Measured by 18FDG-PET/CT Imaging. Chest.

