# Supplementary material for: A Metabolomics Approach to Sulforaphane Efficacy in Secondhand Smoking-Induced Pulmonary Damage in Mice
Source: Metabolites. 2022 Jun 3;12(6):518. doi: 10.3390/metabo12060518 (PMC9227370; doi:10.3390/metabo12060518)
Supplement: Supplementary file 1 [file metabolites-12-00518-s001.zip › metabolites-1701025-Supplementary Figures.pptx]

## Slide 1
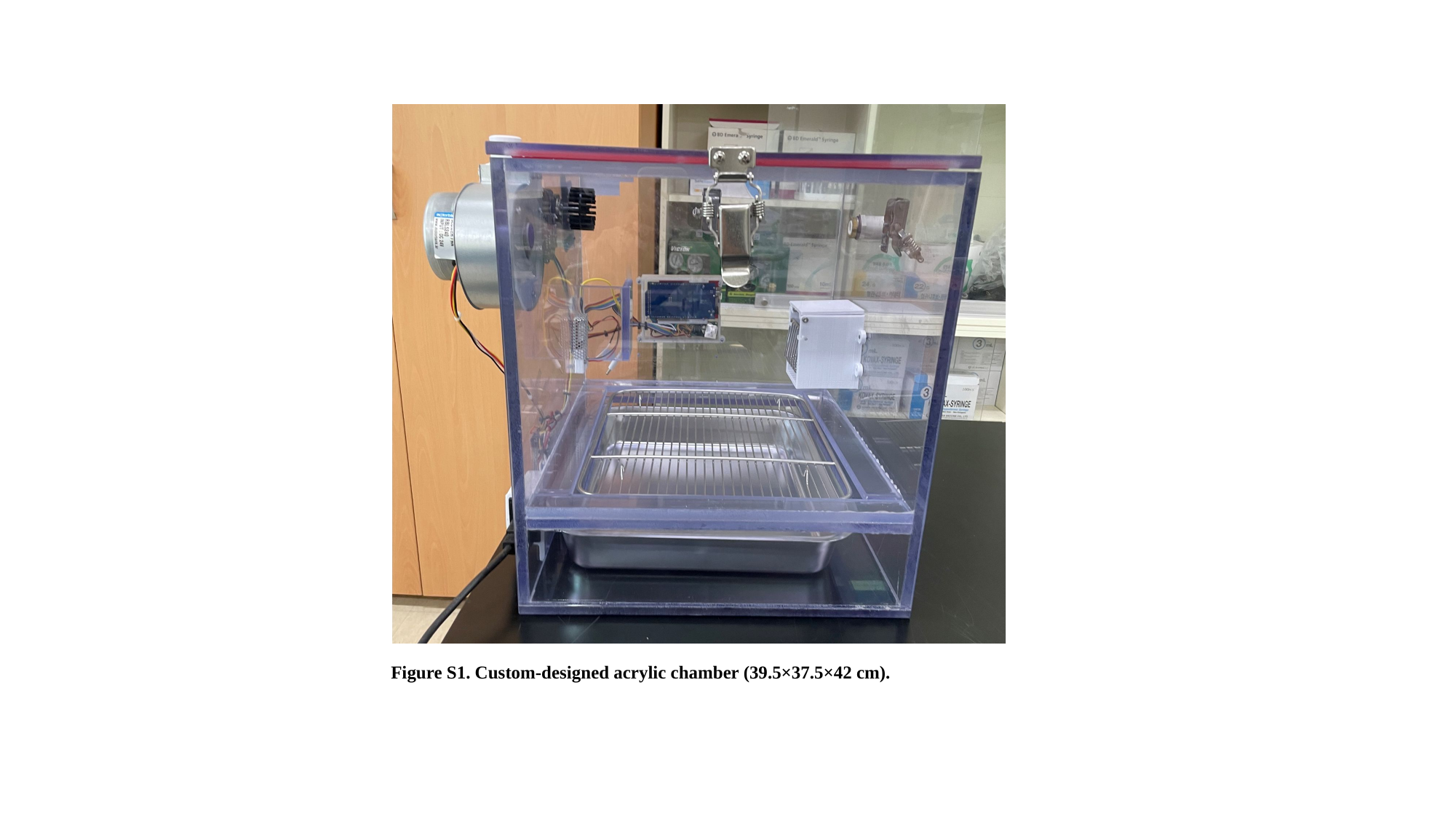

Figure S1. Custom-designed acrylic chamber (39.5×37.5×42 cm).

## Slide 2
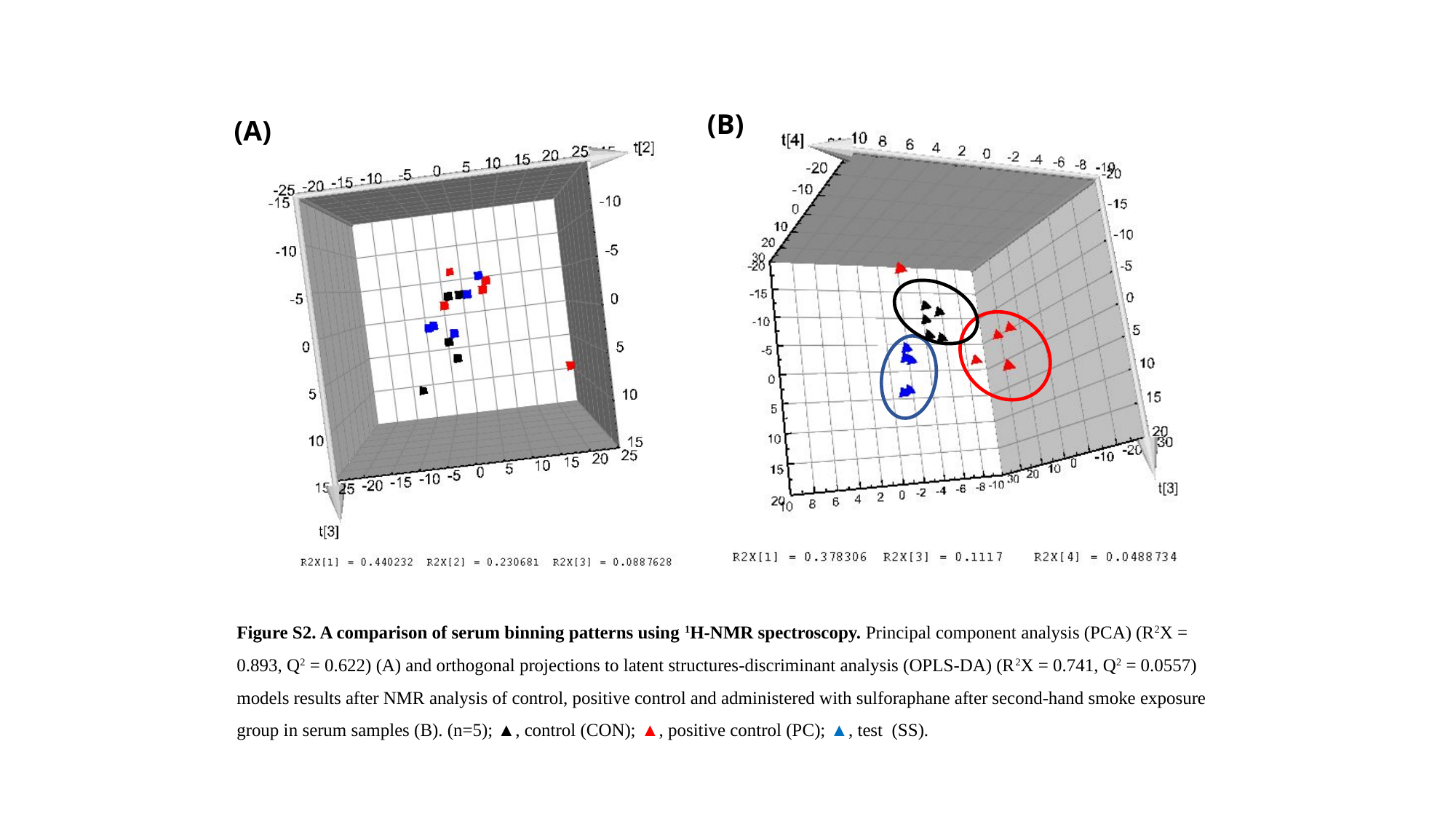

(B)
(A)
Figure S2. A comparison of serum binning patterns using 1H-NMR spectroscopy. Principal component analysis (PCA) (R2X = 0.893, Q2 = 0.622) (A) and orthogonal projections to latent structures-discriminant analysis (OPLS-DA) (R2X = 0.741, Q2 = 0.0557) models results after NMR analysis of control, positive control and administered with sulforaphane after second-hand smoke exposure group in serum samples (B). (n=5); ▲, control (CON); ▲, positive control (PC); ▲, test (SS).

## Slide 3
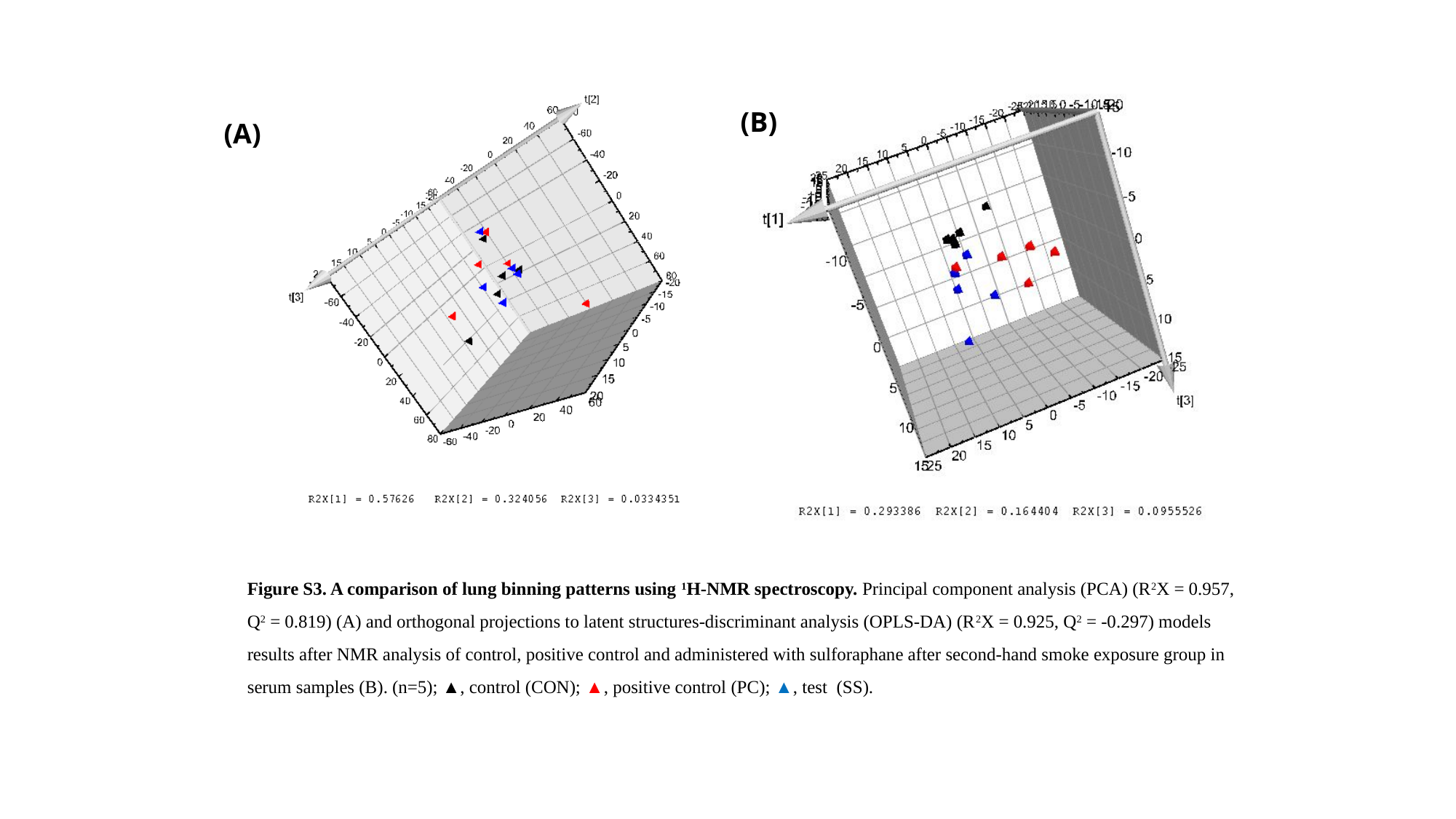

(B)
(A)
Figure S3. A comparison of lung binning patterns using 1H-NMR spectroscopy. Principal component analysis (PCA) (R2X = 0.957, Q2 = 0.819) (A) and orthogonal projections to latent structures-discriminant analysis (OPLS-DA) (R2X = 0.925, Q2 = -0.297) models results after NMR analysis of control, positive control and administered with sulforaphane after second-hand smoke exposure group in serum samples (B). (n=5); ▲, control (CON); ▲, positive control (PC); ▲, test (SS).

## Slide 4
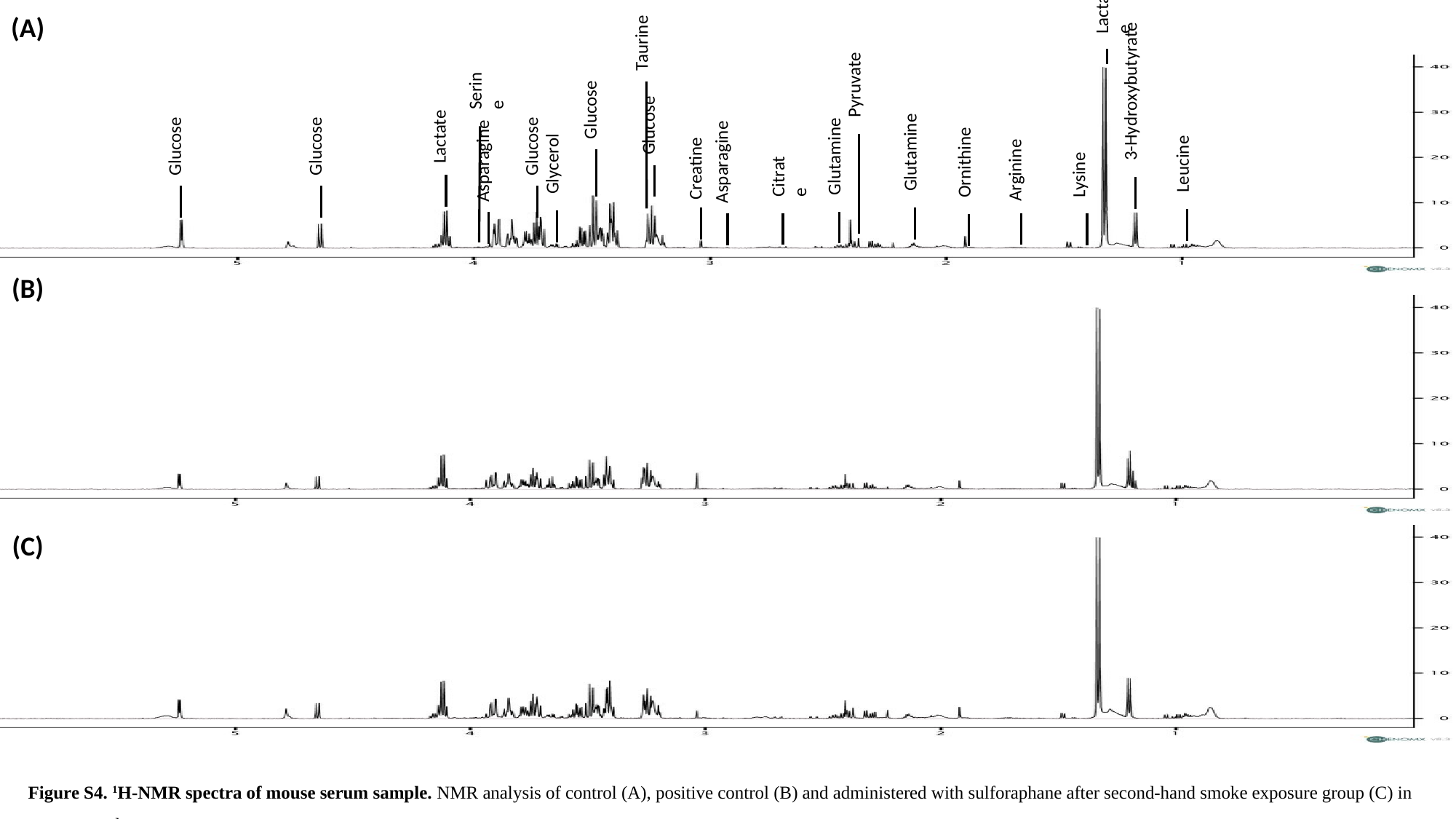

Lactate
3-Hydroxybutyrate
Pyruvate
(A)
Taurine
Serine
Glutamine
Glutamine
Glucose
Leucine
Glucose
Glycerol
Ornithine
Lactate
Creatine
Glucose
Glucose
Glucose
Asparagine
Asparagine
Arginine
Citrate
Lysine
(B)
(C)
Figure S4. 1H-NMR spectra of mouse serum sample. NMR analysis of control (A), positive control (B) and administered with sulforaphane after second-hand smoke exposure group (C) in serum samples.

## Slide 5
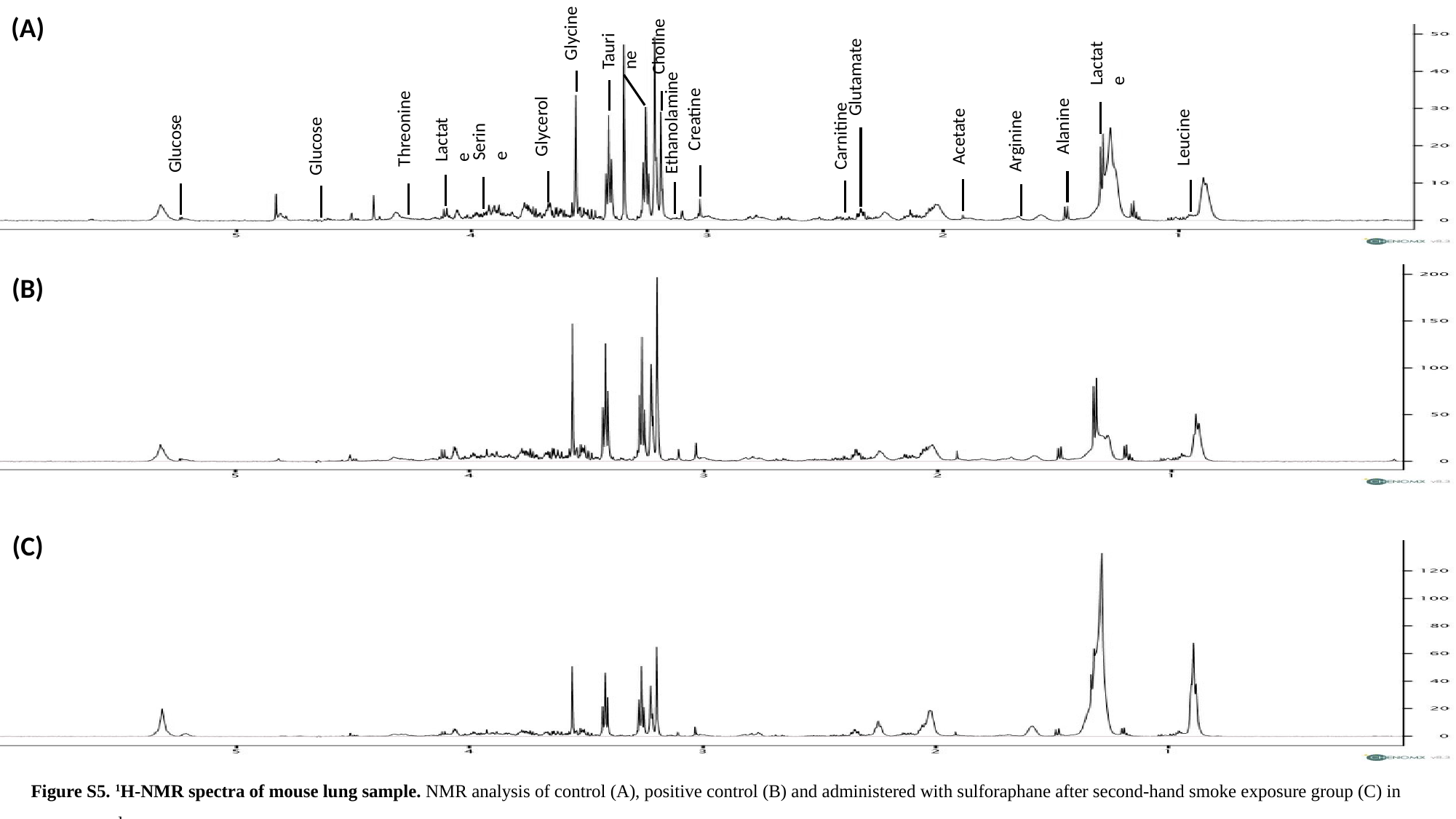

(A)
Glycine
Taurine
Choline
Lactate
Glutamate
Threonine
Leucine
Ethanolamine
Creatine
Glycerol
Alanine
Carnitine
Serine
Lactate
Acetate
Arginine
Glucose
Glucose
(B)
(C)
Figure S5. 1H-NMR spectra of mouse lung sample. NMR analysis of control (A), positive control (B) and administered with sulforaphane after second-hand smoke exposure group (C) in serum samples.
